# Advection, diffusion and linear transport in a single path-sampling Monte-Carlo algorithm: Getting insensitive to geometrical refinement

**DOI:** 10.1371/journal.pone.0330604

**Published:** 2025-09-12

**Authors:** Loris Ibarrart, Stéphane Blanco, Cyril Caliot, Jérémi Dauchet, Simon Eibner, Mouna El Hafi, Olivier Farges, Vincent Forest, Richard Fournier, Jacques Gautrais, Raj Konduru, Léa Penazzi, Jean-Marc Trégan, Thomas Vourc’h, Daniel Yaacoub

**Affiliations:** 1 Université de Toulouse, Mines Albi, UMR- Centre RAPSODEE, Campus Jarlard, Albi CT Cedex 09, France; 2 LAPLACE, Université de Toulouse, CNRS, INPT, UPS, Toulouse, France; 3 Centre National d’Etudes Spatiales, Toulouse, France; 4 PROMES - CNRS, UPR, Odeillo, France; 5 CNRS, UPPA, E2S, LMAPAllée du Parc Montaury, Anglet, France; 6 Université Clermont Auvergne, Clermont Auvergne INP, CNRS, Institut Pascal, Clermont-Ferrand, France; 7 Université de Lorraine, CNRS, LEMTA, Vandœuvre-lès-Nancy, France; 8 Méso-Star, Longages, France; 9 CRCA, CBI, Université de Toulouse, CNRS, Toulouse, France; University of Lakki Marwat, PAKISTAN

## Abstract

We address the question of numerically simulating the coupling of diffusion, advection and one-speed linear transport, with a specific focus on managing geometrical complexity. We base our work on recent advances from the computer graphics community, which has developed Monte Carlo algorithms simulating linear radiation transport in physically realistic scenes, with numerical costs that remain unaffected by geometrical refinement: adding more details to the scene description does not impact the computation time. The resulting benefits in terms of engineering flexibility are already fully integrated into the cinema industry and are gradually being adopted by the video game industry. Here we demonstrate that the same insensitivity to the geometric complexity can be achieved when considering not only one-speed linear transport, but also its coupling with diffusion and advection. In this case, pure linear-transport paths are replaced with advection-diffusion/linear-transport paths, which are composed of subpaths. Each subpath represents one of the three physical phenomena, and coupling is handled by switching from one subpath (i.e. phenomenon) to another. This approach is illustrated using a porous medium involving up to 10,000 pores, with the computation time being strictly independent of the number of pores, showing its ability to facilitate engineering calculations in complex geometries.

## Introduction

Applicative contexts are numerous where diffusion, advection and linear transport physics interact within systems requiring refined geometric descriptions. Engineers attempting to closely understand and optimize the transfer of heat by conduction, convection and radiation in electronic and electric devices of increasing powers and decreasing sizes is among the most highlighted examples, but complexifying the geometric description is also a major requirement in domains such as housing insulation, energy reception in concentrated-solar plants, process optimization in steel industry, etc. Beyond heat transfer, similar needs are associated with many types of transport physics, e.g. neutron transport for nuclear reactors design and operation, charge carriers transport in solar cells *etc*. In all such contexts, numerical simulations impose constraints in terms of geometrical description, which engineers translate into trade-offs between accuracy and computer time or power. There is therefore a strong demand for numerical approaches that know no limit in terms of geometrical refinement.

When only linear transport is considered, it can be stated that the problem is at least partly solved: after two decades of research on acceleration techniques for path-tracing Monte Carlo algorithms, today’s film industry renders highly complex scenes with such an ease that they free the artists from all previous constraints. Scenes can be conceived without worrying about how the rendering algorithms will behave: it is acted that the impact of geometry on accuracy and calculation times is very low. Physicists have successfully adapted and even upgraded these tools to deal with other types of linear transport questions, e.g. for solar and infrared radiative heat transfer in planetary atmospheres where the simulation of 3D multiple scattering in cloudy scenes is now strictly insensitive to the degree of detail of cloud volume and ground surface descriptions [1]. In other applicative fields, the same kind of approach has made it possible to deal with the spectral complexity involved in the Earth’s global radiative cooling [2], or radiative properties of complex-shaped scatterers [3].

We address the same need to deal with one-speed linear transport coupled with diffusion and advection. This implies that

1. new paths are defined to account for the coupling of these three physics;
2. these paths preserve the features that allow path-tracing acceleration in computer graphics.

For pure linear transport, path-sampling Monte Carlo algorithms are designed using the available path-integral formulation of the general solution of the linear Boltzmann equation, which is possible whatever the geometry. It is actually quite straightforward to switch from this specific partial differential equation (PDE) to an integral over particle-paths, where a path is a succession of continuous lines (straight lines for linear transport in the absence of external force, or for radiation in the absence of refraction effect) interrupted at collision events (absorption and scattering within the volume, absorption and reflection at the boundary). These paths will here be called *linear-transport paths*.

Advecto-reacto-diffusive PDEs can be handled similarly using the Feynman-Kac formula [4], which gives the exact solution as the expectation of an Itô process driven by a stochastic differential equation (SDE) [5]. The Itô process being Markovian, Monte-Carlo techniques can be used to solve these PDEs by simulating random paths of the stochastic process, the same way linear-transport paths are sampled to solve the linear Boltzmann equation. Feynman-Kac formula handles a first level of coupling: the physics of advection, reaction and diffusion within a unique geometrical domain are handled using paths that will here be called *advecto-diffusive paths* (or *shifted-brownian paths*). The name of these paths does not include reaction because reaction occurs either at all location along the path, or at the beginning and the end of each path (depending on the formulation): the paths themselves only translate advection and diffusion. In our case, if the Boltzmann equation solution were known at all location within the volume and at the boundary, then linear transport would enter the description via such a reactive term and the advecto-diffusive paths would transport the corresponding sources throughout the domain as expected from Feynman’s vision of propagative processes, in strict correspondence with Green formalism.

In the applicative contexts listed above, the coupling goes further in three directions:

- in the advecto-reacto-diffusive equation, sources that obey Boltzmann equation are unknown,
- these sources depend on the solution of the advecto-reacto-diffusive equation itself, since Boltzmann equation includes source terms obeying the advecto-reacto-diffusive equation,
- the domain is typically divided into subdomains where there is no advection and no contribution of linear transport physics (solids in the following) and subdomains with all three physics interacting (fluids in the following).

We therefore need a formalism in which advecto-diffusive paths interact with linear-transport paths, and also interact with pure diffusive paths at the interface between subdomains (depending on the retained physics, typically ensuring continuity of the flux density when combining solid-diffusion at one side and fluid-diffusion at the other side, and linear transport particles emission/absorption at the interface itself). This question was already addressed by several authors with the objective of simulating the transfer of heat in 3D porous media. [6,7] and [8] dealt with thermal diffusion inside opaque solids interacting via radiation, with also convective heat transfer along the solid surfaces for a fluid at a known temperature: advection and diffusion within the fluid is not part of the simulation. [9] adds the simulation of convection within fluid cells that are assumed to be perfectly mixed, which means that all three heat transfer modes are coupled but the fluid modeling does not require advection. Among these researches, [10] has begun to formulate the corresponding path integrals, using a double-randomization approach, introducing paths that are recursively defined as successions of sub-path corresponding to each of the three heat transfer modes, still with a convection model requiring no advection, and only at the stationary limit. The theoretical foundations that make this coupling possible are fully exposed in [11]. This approach has been successfully implemented in [12] and [13], showing its ability to solve such coupled problems in a complex environment.

Here, we will make use of the same formalism for problems involving pure diffusion in some parts of the geometry (solids), advecto-diffusion in the remaining part (fluid) and one-speed linear transport within the fluid and between the solid-fluid interfaces (exchanges between fluid volumes, between a fluid volume and the solid surfaces, and between the solid surfaces through the absorbing and scattering fluid). This will allow us to test the corresponding advection-diffusion/linear-transport path-sampling Monte Carlo algorithms on realistic configurations inspired by porous solid-fluid heat exchangers.

In terms of preserving the features that make path-tracing acceleration efficient in computer graphics, one-speed linear transport can be treated exactly like radiative transfer in computer graphics and the recursive switching from one sub-path to the next one is independent of the detail of the geometrical description. The question therefore reduces to the acceleration of advecto-diffusive paths within a geometrical domain defined using a very large number of geometric primitives.

The ability to produce strict reformulations in path space for models initially formulated in differential form is a difficult and still largely unresolved issue, especially when there are heterogeneities in thermophysical properties and domain boundaries with prescribed boundary conditions. In addition, it is necessary to probabilize these formulations to produce unbiased estimators of the solution following the Feynman-Kac approach.

These formulations rely on defining underlying stochastic processes that must be sampled exactly to produce unbiased estimators of the solution. For instance, in the case of a standard Brownian process in an infinite field, it is straightforward to sample the positions and times of a particle without knowing its entire trajectory. Similarly, since the 1970s, proposals have been made to represent unbiased Brownian motions within confined domains, mainly involving methods based on Green’s function formulations in terms of position and first-passage time in highly symmetrical domains (spheres, parallelepipedes, etc.) [14–16]. The task becomes significantly more complex if we consider the trajectories that start from the boundaries, and numerous questions remain about the ability to produce unbiased sets of positions and times compatible with the underlying stochastic processes. These questions have been extensively studied by the applied mathematics community working on Monte Carlo methods [17–20], and, more recently, by the computer graphics community [21,22]. From all this work, it emerges that each situation resolved, even with a reduced level of applicability, is a victory. The issue is not only to reformulate the solutions in the formalism of surface integrals but also to adopt a probabilistic perspective that maintains all the physical representations of the underlying paths. Recent work by Rohan Sawhney and co-authors has opened up many new perspectives by proposing unbiased formulations for solving Poisson equations in complex three-dimensional environments with Neumann or Robin boundary conditions [23,24]. However, there is currently no equivalent work that comprehensively addresses the resolution of coupled advecto-reacto-diffusive equations as those presented in the present study. Another constraint of these methods is that they require the knowledge of the closest distance to the boundary, which is typically missing information when using the data structuring and access choices underlying current advanced acceleration strategies (hierarchical grids added on top of unstructured sets of geometrical primitives).

For these reasons, we are compelled to introduce a numerical parameter (discrete walk step and reinjection step), which requires only the knowledge of the line-primitive intersection, the computation of which takes benefits from the path-tracing acceleration tools developed by the computer graphics community. In doing so, it adds computational constraints since the step must be adapted to simulation conditions in order to balance accuracy and computation time, as is the case with all conventional numerical methods.

Below, we will construct an advecto-diffusive path sampling procedure as successions of straight-line jumps. This procedure may not be the best choice for long-term perspectives but it is perfectly suited to available computer-graphics acceleration techniques. We will illustrate how such path-sampling Monte Carlo strategies have the same features as for pure linear transport: computation time is insensitive to the number of geometrical primitives.

In preparation for this illustration, Sect 1 introduces the physical model and Sect 2 shows how the coupling of two physics is translated into the recursive sampling of subpaths, each dedicated to a single physics. An approximate brownian walk is then introduced in Sect 3, which recovers all the features of linear-transport paths as far as tracking acceleration is concerned. Finally Sect 5 provides simulation examples for a configuration emblematic of the heat transfer literature: a porous heat exchanger composed of an increasing number of kelvin cells.

## 1 Model

We consider a motionless solid domain $\Omega_{S}$ of boundary $\partial \Omega_{S}$ (only diffusion, no advection, no linear transport) and an adjacent absorbing and scattering fluid domain $\Omega_{F}$ of boundary $\partial \Omega_{F}$ (all three processes active at all locations). The physical properties are uniform within each part, the velocity v≡v(x) is known at each location x within the fluid, and we act on the geometrical refinement of the geometry by increasing the characteristic scale ratios of $\partial \Omega_{S}$ and $\partial \Omega_{F}$ (smallest physically significant scale divided by system scale), typically by increasing the number of pores in a porous geometry.

For the pure diffusion part and the advection-diffusion part of the model, the physical observable is noted $\eta_{S}\equiv \eta_{S}(x)$ for each location x within the solid ($x\in \Omega_{S}$) and $\eta_{F}\equiv \eta_{F}(x)$ for each location within the fluid ($x\in \Omega_{F}$). For the one-speed linear transport part, the physical observable is noted f≡f(x,u) at each location $x\in \Omega_{F}$ and each direction ***u*** of the unit sphere (noted "4π"). In pure theoretical terms, *η* and *f* can be interpreted as the density and the distribution function of two interacting species. The interaction between the two species is compatible with an equilibrium state where *f* is isotropic:

- the emission direction is isotropically distributed; the corresponding emission rate is $\frac{1}{4\pi }$ times the diffusing species absorption (or reaction) rate $\nu_{a}\;\eta_{F}$,
- the absorption (or reaction) frequency $\nu_{a}$ is the same for both the diffusing species and the linear transport species.

An example of how to translate this model for heat-transfer applications is provided in Appendix F.

**Fig 1:**
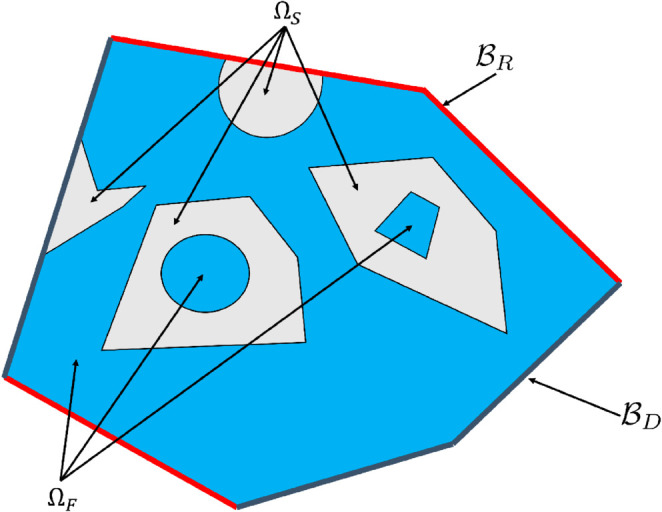
Illustration of the advection, diffusion and linear transport configuration studied in the article. The solid domain $\Omega_{S}$ is shown in gray, the fluid domain $\Omega_{F}$ is shown in blue. The model is: pure diffusion in the solid; advection-diffusion in the fluid; linear transport in the absorbing and scattering fluid. The boundary $ℬ={ℬ}_{D}\cup {ℬ}_{R}$ of the system is the union of ${ℬ}_{D}$, shown in grey, where Dirichlet boundary condition are formulated (the density *η* is prescribed), and ${ℬ}_{R}$, shown in red, where the diffusion flux is null. The incident distribution function *f* obeying linear transport is prescribed over the whole boundary ℬ. See a practical implementation of this generic configuration in Figs 5 and 6.

The boundary of the system is noted ℬ, i.e. $ℬ=(\partial \Omega_{S}\cup \partial \Omega_{F})-(\partial \Omega_{S}\cap \partial \Omega_{F})$. At each location y on ℬ, the incoming distribution function *f*_*i*_ is known, i.e. $f(y,u)=f_{i}(y,u)$ for all u.n(y)>0 where n(y) is the inward normal to the boundary at y. For the diffusive species at the boundary, we note $\eta_{B}(y)$ the value of *η* at each boundary location and ℬ is split in two parts, $ℬ={ℬ}_{D}\cup {ℬ}_{R}$: on ${ℬ}_{D}$ we use a Dirichlet boundary condition ($\eta_{B}$ is known), on ${ℬ}_{R}$ the diffusion flux is null (∇η.n=0). At the interface $\partial \Omega_{S}\cap \partial \Omega_{F}$ between the solid and the fluid, we write the flux continuity for the two species: the diffusion flux on the solid side, $-D_{S}\nabla \eta_{S}.n$, equals the diffusion flux on the fluid side, $-D_{F}\nabla \eta_{F}.n$, plus the linear-transport flux, $j_{T}.n$ where $j_{T}=\int_{4\pi }fcu\;du$. There is no advective flux because velocity is assumed to be null at the solid-fluid interface and density is assumed to be continuous through the interface (Fig 1).

Altogether the model providing $\eta_{S}$, $\eta_{F}$ and *f* in the domains $\Omega_{S}$ and $\Omega_{F}$ is



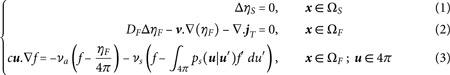



where *D*_*S*_ and *D*_*F*_ are the solid and fluid diffusion coefficients, *c* is the particles speed in the linear transport model, $\nu_{a}$ is the absorption frequency, $\nu_{s}$ is the single-scattering frequency, *p*_*s*_ is the single-scattering phase function and $f^{\prime }\equiv f(x,u^{\prime })$.

Still within the system, at the solid-fluid interface, we have:



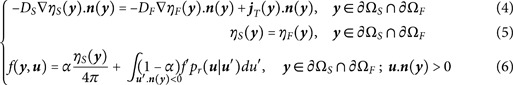



where *α* is the surface absorptivity (*i.e.* one minus reflectivity) and *p*_*r*_ the probability density of the direction after surface reflection.

At the boundary of the system, we have:



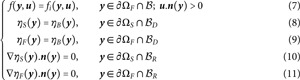



Note that Robin boundary conditions would make no difference as far as the present numerical discussion is concerned.

## 2 Interacting random walks

Monte Carlo algorithms solving Eqs 1, 2 and 3, each separately, are well established. They all start with the definition of a random process, with the solution of the addressed model shown as the expectation of this process. For Eq 1 (pure diffusion), the process is brownian motion. Regarding Eq 2, we consider from Eq 3 that $-\nabla .j_{T}=-\nu_{a}\left(\eta_{F}-\int f\;du\right)$ has the form of a standard reaction term provided that Eq 2 is decoupled, i.e. provided that *f* is known. Hence, Eq 2 is diffusion-advection-reaction, and, in accordance with Feynmann-Kac theory, the process is an exponentially interrupted brownian motion with drift. For Eq 3 (linear Boltzmann equation), the process is a backward multiple-scattering and reflection walk. The remaining question is therefore coupling.

When coupling is linear, the main idea behind the combined use of two Monte Carlo algorithms is double randomization [25,26]. If

- a Monte Carlo algorithm is available for solving problem 1 with *N* samples of process P1,
- sampling P1 requires the solution of problem 2,
- a Monte Carlo algorithm is available for solving problem 2 with *N* samples of process P2,

there is no need to sample P2 *N* times each time it is needed for P1, which would require N×N samples: it suffices to use a single sample of P2. If, for instance, P1 is an interrupted brownian motion that requires the solution of P2 at the interruption location, and if P2 is a backward multiple-scattering walk, then the overall process, representing the coupling of the two underlying physics, is simply a brownian motion that, when interrupted, switches to a multiple-scattering path. This remains meaningful when the coupling is both ways, i.e. sampling P2 requires the solution of problem 1. This is the case when addressing the coupling of Eq 2 and Eq 3: when interrupted, the brownian motion switches to a multiple-scattering path (because the reaction term involves *f* and it is unknown), and when this multiple-scattering path is interrupted at an absorption/emission location, it switches back to brownian motion (because the emission term involves $\eta_{F}$ and it is unknown).

**Fig 2:**
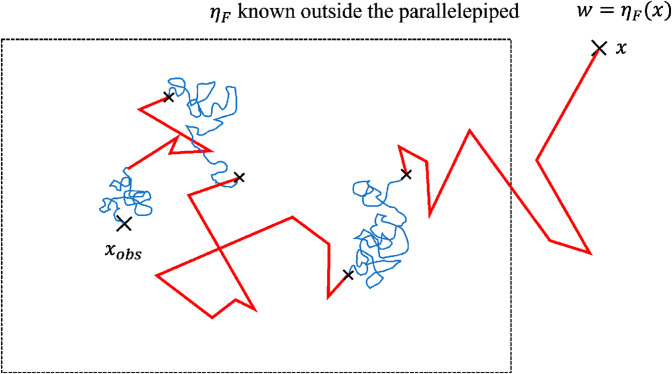
A diffusive/linear-transport path in an infinite uniform static fluid for which the density $\eta_{F}$ is known outside a rectangular cuboid. *N* such paths are sampled to estimate $\eta_{F}(x_{obs})$. Each path starts from the observation position $x_{obs}$ and returns a Monte Carlo weight equal to the value $\eta_{F}(x)$ at the location x of the first brownian-walk interruption occurring outside the parallelepiped. $\eta_{F}(x_{obs})$ is estimated as the average of these *N* weights. Blue sub-paths are brownian walks. Red sub-paths are multiple-scattering linear-transport paths. All algorithmic details are provided in the text of Sect 2. Simulation examples are provided in Appendix A and Table 1.

Fig 2 illustrates the practice of such a combination of brownian paths and multiple-scattering linear-transport paths in the simplified case of an infinite uniform static fluid. The resulting Monte Carlo algorithm evaluates $\eta_{F}(x_{obs})$ at a given location $x_{obs}$ assuming that $\eta_{F}$ is known at a given distance from $x_{obs}$. It starts with the brownian walk by setting the initial location $x_{B}$ to $x_{obs}$ and the initial time *t*_*B*_ to *t*_*obs*_. The first step is then the sampling of an exponentially distributed duration $\delta t_{B}$ for the brownian walk before interruption by emission. The expectation of this exponential distribution is $\frac{1}{\nu_{a}}$. Then the location of the brownian walker at $t_{B}\;+\;\delta t_{B}$ is sampled according to a three-dimension gaussian distribution centered on $x_{B}$ of standard deviation $\sigma =\sqrt{6D\delta t_{B}}$, where *D* is the diffusion coefficient of the medium. At this new location $x_{T}$, a linear-transport path is initiated, which starts with the isotropic sampling of a direction ***u***. Then the algorithm is a standard multiple-scattering algorithm:

- a free-flight duration $\delta t_{T}$ is sampled according to an exponential distribution of expectation $\frac{1}{\nu_{a}\;+\;\nu_{s}}$
- the multiple-scattering path is started with a straight line from $x_{T}$ to $x_{T}\;+\;c\delta t_{T}u$
- at this collision location, a test is made to decide between absorption and scattering
- if scattering is retained, a new direction $u_{s}$ is sampled according to *p*_*s*_ and the linear-transport path continues in this new direction.

The linear-transport path ends when absorption is retained, and at this absorption location a new brownian walk is started. This creates a succession of a brownian walk, a multiple-scattering path, a brownian walk, etc, until one of the multiple-scattering path is interrupted at a location $x_{B,i}$ where $\eta_{F}$ is known. The Monte Carlo weight *w* is $w=\eta_{F}(x_{B,i})$ and the Monte Carlo estimate is the average of *N* such weights. In this simple configuration, both the brownian and linear-transport walks can be sampled exactly and the Monte Carlo estimate is strictly unbiased.

The fact that the estimate is strictly unbiased in this example will not be preserved in the remainder of this article. This is due to the interface and boundary conditions that will lead to brownian walks in confined domains: we will handle these confined walks via a numerical approximation that will introduce a bias. But appart from this approximation (that reduces to zero when lowering the numerical parameter to zero), it is exactly the same approach that will be used hereafter for the coupling of linear transport with diffusion and advection. In particular, the computation times indicated in Table 1 illustrate a well known feature of Monte Carlo simulations that we will also observe in all the forthcoming simulation examples: in the low Knudsen number regime (or high optical thickness regime), i.e. when scattering widely dominates absorption and the mean free path is very small compared to the dimension of the system, then a large number of scattering events are required before either the linear-transport walk is interrupted to initiate a brownian walk, or it reaches the limit of the domain. This feature will not vanish with the following developments: when we will illustrate an insensitivity to the complexification of geometry, this will only mean that the refinement of the geometrical description has no impact on the computation time requirements, but these requirements will remain direct functions of the single-scattering albedo and Knudsen number.

**Table 1:** Simulation results for the diffusion/linear-transport algorithm of Sect 2 with the parameters of A. $L^{3}\tilde{\eta_{F}}(x_{obs})$ is the estimation of $L^{3}\eta_{F,exact}(x_{obs})=2$ that is provided by *N* = 10^5^ Monte Carlo samples and *σ* is its associated statistical uncertainty as provided by the standard error (take 3σ for a 0.9995 confidence). *T* is the computation time recorded when performing the *N* = 10^5^ Monte Carlo samples, $N_{1\%}$ is the required number of Monte Carlo samples in order to reach $\sigma =0.01\eta_{F,exact}(x_{obs})$ and $T_{1\%}$ is the associated computation time. Computations were made using a x86i_64 Intel(R) Core(TM) i9-9880H CPU 2.30GHz.

$\frac{c}{\nu_{s}L}$	$\frac{D_{F}}{cL}$	$L^{3}\tilde{\eta_{F}}(x_{obs})$	$L^{3}\sigma$	*T*(*s*)	$N_{1\%}$	$T_{1\%}(s)$
100	0.01	2.001	0.004201	0.6247	4411	0.02756
100	1.	1.990	0.006390	0.6897	10207	0.07040
100	100.	1.927	0.05176	0.6919	669758	4.634
1.	0.01	1.999	0.003586	0.7919	3214	0.002546
1.	1.	1.993	0.006148	0.6196	9450	0.05855
1.	100.	1.927	0.05165	0.6028	666982	4.021
0.01	0.01	2.000	0.001850	99.04	855	0.8476
0.01	1.	2.004	0.005765	16.37	8309	1.360
0.01	100.	2.020	0.05145	13.16	661672	87.10

## 3 Brownian walks in confined domains

The work of Feynmann and Kac in the 50’s [4] played a key role in establishing the links between parabolic partial differential equations and random processes. Feynmann-Kac’s formula states that the solutions of advection-diffusion-reaction equations (Eqs 1 and 2 in our context) can be rewritten as expectation of stochastic processes. Together with the usual translation of linear transport theory in statistical terms (for Eq 3), this is all the theoretical basis required to justify Monte Carlo algorithms such as the one illustrated in the previous section. However, this statement leaves aside the huge amount of theoretical and numerical work that was required to deal with boundary conditions, before such algorithms became practical.

The family of Random Walk on Sphere (RWS) algorithms dominates the corresponding literature. The starting point is the observation that exact sampling of confined brownian walks is only possible for simple geometries and then the question becomes the following: how is it possible to cover any geometry using simple patterns such as spheres, sample exact brownian paths inside these patterns in a computationally efficient way, make numerical approximations in the vicinity of the boundaries (any boundary cannot be adjusted exactly with a sphere for instance), and ensuring that the parameters of these approximations can be tuned to reach the required accuracy (ensuring convergence with computational costs remaining satisfactory)?

The historic RWS method proposed by Brown in [27], and rigorously justified by Muler in [28], makes use of the distribution of first passages at the sphere, for a brownian motion starting from the center of the sphere: this distribution is uniform. Using the same logic as in the previous section, this allows to write the solution of the stationary diffusion equation as the expectation of the density at random locations on the sphere (which is also the harmonicity property of the laplacian operator). In the present context, this solves Eq 1 at the center of any sphere as soon as $\eta_{S}$ is known at all locations on the sphere:

$$\eta_{S}(x)=\int_{4\pi }\frac{1}{4\pi }du\;\eta_{S}(x\;+\;\delta u),\;x\in \Omega_{S},\;\delta ⩽\max_{\tilde{\delta }}(\{𝒮(x,\tilde{\delta })\cap \partial \Omega_{S}\}=\emptyset )$$ (12)

where $𝒮(x,\tilde{\delta })$ is the sphere of radius $\tilde{\delta }$ centered at x. If $\eta_{S}$ is not known at the location x+δu sampled on the sphere, then double randomization is used with a new sphere centered on this new location, etc. The system boundary is then treated as follows:

- the sphere is chosen as the largest sphere entirely within the domain (the radius is the closest distance to the boundary, *i.e.* $\delta =max_{\tilde{\delta }}(\{𝒮(x,\tilde{\delta })\;\cap \;\partial \Omega_{S}\}=\emptyset )$) and when the location sampled on the sphere is at a distance lower than a numerical parameter *ε*, then the random walk is considered to have reached the boundary;
- the corresponding approximation was very deeply investigated and convergence is satisfied, the number of successive spheres required to reach the boundary increasing only as the logarithm of $\frac{1}{\epsilon }$ when reducing *ε*.

**Fig 3:**
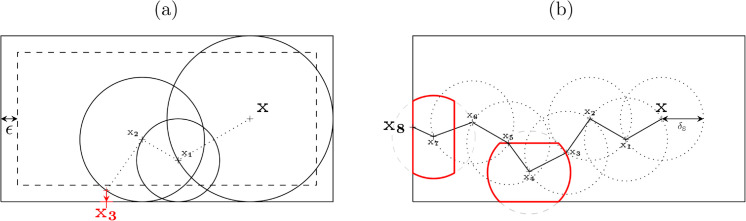
Sampling of confined brownian walks to estimate the density *η* at point x. (a) Using the RWS method: the next positions $x_{i}$ are sampled on the largest sphere inscribed in the domain until the sampled position is on the boundary, where *η* is known. In order to end the algorithm, the studied domain’s surface is thickened by a small value *ε* in which the position is projected on the border. (b) Using the proposition made in Sect 4, that is compatible with ray tracing. All the spheres have the same radius $\delta_{S}$. When getting close to a boundary, the displacement is truncated through the knowledge of the distance to the surface in this direction, which make the spheres distorded.

The corresponding algorithm used to evaluate the density at any probe position $x_{obs}$ is showcased in Fig 3a.

This proposition has been extended in numerous directions, notably with walks on rectangular parallelepipedes [16]. Considering the present context, we can briefly highlight the fact that drifted brownian walks can be handled very similarly. For instance, Sabelfeld [29] used first passage statistics of drifted brownian motion in a sphere to extend the RWS method to couple advection and diffusion for cathodoluminescence and electron beam induced current imaging. At the stationary state, for a homogeneous velocity field, it was proven that the distribution of first passage positions in a sphere corresponds to the von Mises-Fisher distribution. Eq 13 shows how this distribution can be applied to Eq 2. It can be noted that in the case of a zero velocity field, this expression leads to Eq 12, and to a Dirac distribution centered in v=u|v| at the limit of an infinite local Peclet number. For Eq 2 without coupling with linear transport,

$$\eta_{F}(x)=\int_{4\pi }p_{MF}(u|v)du\;\eta_{F}(x\;+\;\delta u),\;x\in \Omega_{F},\;\delta ⩽\max_{\tilde{\delta }}(\{𝒮(x,\tilde{\delta })\cap \partial \Omega_{F}\}=\emptyset )$$ (13)

with the probability density of von Mises-Fisher distribution


$$p_{MF}(u|v)=\frac{1}{4\pi }\;\frac{\frac{Pe}{2}}{\sinh\left(\frac{Pe}{2}\right)}\exp\left(-\frac{Pe}{2}\frac{v.u}{\left|v\right|}\right)$$

where $Pe=\frac{\delta |v|}{D_{F}}$.

When refining the geometrical description, the main point in Eqs 12 and 13 is the fact that each step requires the computation of the local closest distance to the boundary (to ensure $\delta =max_{\tilde{\delta }}(\{𝒮(x,\tilde{\delta })\cap \partial \Omega_{S}\}=\emptyset$). Accessing this information is a crucial issue, as it can be computationally very demanding, and it is repeated at each step along the path. Although very significant research efforts are devoted to this question of accessing the shortest *sphere-surface intersection* [30,31], the costs of today’s algorithms are still much higher than those of the algorithms accessing the shortest intersection with the geometry for a straight line in a given direction (*line-surface intersection*). The latter ones have indeed been deeply investigated by computer graphics community because it is required by ray tracing, a method commonly used to model light transport. This research has produced acceleration techniques based on precomputed recursive grids that are now practically available for common usage by numerical scientists [32,33]. Hence, at the present stage of these researches, the insensitivity to geometrical refinement illustrated in [1] for radiative transfer, is directly connected to the fact that accessing the geometry is reduced to finding line-surface intersections and not sphere-surface intersections. This can be amended in the future. In this case we get ready to adapt to these potential new tools. Still, the main idea of couplings subpath through double-randomization (see Sect 2) is unaffected.

## 4 An example proposition compatible with ray-tracing

Before sphere-surface intersection algorithms reach a similar maturity, RWS algorithms must be adapted to match this requirement of only accessing information about the geometry via line-surface intersections. We make here a proposition for an algorithm inspired of the RWS literature complying with this condition, so that acceleration techniques described for instance in [1] will be directly available for a numerical implementation handling highly refined geometries.

This is just one example of a proposition. Alternatives could be formulated, aiming for instance at increasing the convergence order or reducing the computation time associated to the computation of line-surface intersections in the vicinity of the boundary, but they would all lead to the same conclusion as far as the objective of the present article is concerned: insensitivity to geometrical refinement can still be achieved when introducing brownian motion in a confined domain. Each algorithm presented below is detailed in Appendix D. All are validated against available analytical solutions in Appendix E and against numerical solutions in Appendices F.3 and F.4. Differences between our approach and deterministic numerical schemes are also discussed in [34,35] about applications regarding heat transfer.

### 4.1 A finite difference scheme for pure diffusion in the solid

For the handling of geometrical confinement with only line-surface intersections, we made the choice of designing approximate brownian walks as direct statistical translations of standard finite difference or finite volume approaches to advection-diffusion-reaction partial differential equations. Regarding pure diffusion, the formulation of Eq 12 is kept unchanged, but with a fixed step $\delta_{S}$ (instead of the closest distance to the boundary). Therefore, at each step, given a sampled direction ***u*** on the unit sphere (noted 4π), the walk should shift to the position $x\;+\;\delta_{S}u$. However, in order to account for boundaries, the rays defined by (x,u) and (x,−u) are traced; let $\delta_{u}$ and $\delta_{-u}$ be the distances between x and the boundary in the directions ***u*** and −u respectively. Then, the walk is only shifted by the minimum of $\delta_{S}$, $\delta_{u}$, and $\delta_{-u}$, which leads to a position in $\Omega_{S}$ or directly on its boundary $\partial \Omega_{S}$. This algorithm corresponds to Eq 14 (see Fig 3b and Algorithm 2 in Appendix D).

$$\eta (x)=\int_{4\pi }\frac{1}{4\pi }du\;\eta (x\;+\;\delta (x,u)\;u),\;x\in \Omega_{S},\;\delta =\min(\delta_{S},\delta_{u},\delta_{-u})$$ (14)

Under the assumption that the density field is locally trilinear within $𝒮(x,\delta_{S})$, this equation exactly solves Eq 1 (see a demonstration in Appendix B).

### 4.2 A Patankar’s scheme for coupling diffusion with advection and linear transport in the fluid

Regarding coupled advection and diffusion, Patankar’s work [36] suggests a numerical scheme based on the analytical solution of Eq 2 in a 1D homogeneous media. By interpreting the coefficients in Patankar’s scheme as probabilities, we propose an approximate random walk with drift for advection-diffusion. As in Sect 2, coupling with linear transport will be handled thanks to an interruption time at which brownian motion switches to a linear-transport walk. In the present context, this interruption time must preserve all the numerical features inspired by Patankar work, ensuring that, for a uniform velocity field, the walk recovers the available exact solution of an exponential density profile in the velocity direction and a linear profile in the orthogonal plane. This is achieved by introducing a probability *p*_*T*_ to switch to linear transport at each Patankar step. We give all the details of the formal developments leading to these probabilities in Appendix C. In a local orthonormal basis $\left(O,e_{1},e_{2},e_{3}\right)$ chosen such that $v=e_{1}|v|$, it leads to Eq 15 where *δ* is a fixed step and $u_{i}=(-1){\;}^{i}e_{\left\lfloor \frac{i\;+\;1}{2}\right\rfloor }$ (⌊ ⌋ being the floor function, so that $u_{1}=-e_{1}=-v/|v|$ and $u_{2}=e_{1}=-u_{1}$).

$$\eta (x)=p_{T}\;\eta_{T}(x)\;+\;(1-p_{T})\sum_{i=1}^{6}p_{i}\eta (x\;+\;\delta u_{i}),\;x\in \Omega_{F}$$ (15)

with



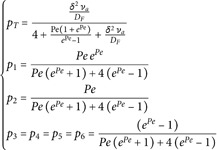

(16)


where $\eta_{T}=\int f\;du$ is the density of the linear-transport species described by Eq 3 and $Pe=\frac{\delta \;v}{D_{F}}$.

**Fig 4:**
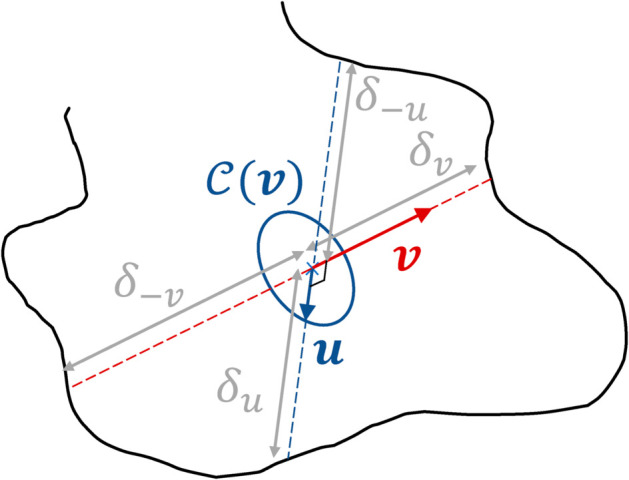
Example of ray tracing to evaluate the length of the next step in the approximate random walk based on Patankar scheme: starting from the current location, two rays are drawn in the velocity direction *v* and its opposite, and two others in a random diffusion direction *u* (and its opposite) in the plane orthogonal to *v.* These four rays enable to compute $\delta =\min(\delta_{F},\delta_{v},\delta_{-v},\delta_{u},\delta_{-u}$).

This equation can be directly interpreted as a random walk, which has the probability 1−*p*_*T*_ to move in 6 directions according to their 6 respective probabilities, and the probability *p*_*T*_ to switch to a linear-transport walk (see Algorithm 3 in Appendix D). The above expression is valid for any orientation of the local orthonormal basis around $e_{1}=v/|v|$. Therefore, Eq 15 can be averaged over the direction $e_{2}$ (and subsequently $u_{3,4,5,6}$), leading to Eq 17. In the algorithmic translation of this equation, at each step of the walk, a diffusion direction ***u*** is uniformly sampled on the unit circle 𝒞(v) in the plane perpendicular to ***v***. This way, when dealing with the boundaries, the diffusion directions can be treated exactly as in Sect 4.1. Following the same guidelines as previously, each step now requires four rays tracing to evaluate the length of the next step: two in the diffusion direction to compute $\delta_{u}$ and $\delta_{-u}$, and two in the velocity direction to compute the distances $\delta_{v}$ and $\delta_{-v}$ between x and the first surface in the directions ***v*** and −v respectively (see Fig 4). These 4 distances $\delta_{v}$, $\delta_{-v}$, $\delta_{u}$, $\delta_{-u}$ are then compared to the fixed step $\delta_{F}$ (that is equivalent to $\delta_{S}$ in the solid).

$$\eta (x)=p_{T}\;\eta_{T}(x)\;+\;(1-p_{T})(p_{1}\;\eta \left(x-\delta \frac{v(x)}{\left||v(x)|\right|}\right)\;+\;p_{2}\;\eta \left(x\;+\;\delta \frac{v(x)}{\left||v(x)|\right|}\right)\;\;+\;4\;p_{3}\int_{𝒞(v)}\frac{1}{2\pi }du\;\eta (x\;+\;\delta u)),\;x\in \Omega_{F},\;\delta =\min(\delta_{F},\delta_{v},\delta_{-v},\delta_{u},\delta_{-u})$$ (17)

where *p*_*T*_, *p*_1_, *p*_2_, and *p*_3_ are defined as mentioned for Eq 16 (but with an updated value of $\delta =\min(\delta_{F},\delta_{v},\delta_{-v},\delta_{u},\delta_{-u})$).

### 4.3 Boundary and interface conditions

Using the same approach as in [37] for the solid-fluid interface, the density gradients in Eq 4 can be approximated by finite differences using the same small steps $\delta_{S}$ and $\delta_{F}$ as in the previous sections. The source term $j_{T}(y)\;\cdot \;n(y)$ in Eq 4 can be expressed as the difference between the incident and emitted flux densities in the hemisphere around the surface normal n(y):

$$j_{T}(y)\cdot n(y)=\frac{\alpha }{4}c\;\eta (y)-\frac{\alpha }{4}c\int_{2\pi }\frac{u\cdot n(y)}{\pi }\;4\pi f(y,-u)\;du$$ (18)

which leads to

$$\eta (y)=p_{S}\eta_{S}(y-\delta_{S}n)\;+\;p_{F}\eta_{F}(y\;+\;\delta_{F}n)\;+\;p_{T_{i}}\int_{2\pi }\frac{u\cdot n(y)}{\pi }\;4\pi f(y,-u)\;du,\;y\in \partial \Omega_{S}\cap \partial \Omega_{F}$$ (19)

where



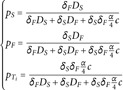

(20)


Again, in Monte Carlo terms, these probabilities are to be understood with regards to the double-randomization concept leading to successive sub-paths, as in Sect 2. When a random walk hits a solid-fluid interface, Eq 19 makes the walk switch to a diffusive walk starting in the solid at $\left(y-\delta_{S}n\right)$ with a probability *p*_*S*_ (see Sect 4.1), an advecto-diffusive walk starting in the fluid at $\left(y\;+\;\delta_{F}n\right)$ with a probability *p*_*F*_ (see Sect 4.2), or a linear-transport walk starting at the point y in a direction −u toward the fluid, distributed according to the Lambertian law of surface emission (or according to an other law if surface emissivity depends on direction).

The same approach is used when dealing with boundary conditions involving a diffusion flux, i.e. Eqs 10 and 11, which leads to a normal reinjection:



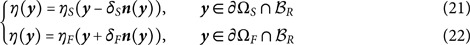



In practice, even if the lengths $\delta_{S}$ and $\delta_{F}$ are supposed small enough, another surface might be present between y and its point of re-injection in the solid or fluid (e.g. in sharp corners). To avoid this problem, two rays are traced from the point y in directions ***n*** and −n to redefine the lengths $\delta_{S}$ and $\delta_{F}$ for this one step only : if a surface is detected between y and its point of re-injection, the length is reduced so that the re-injection point ends up strictly in the midpoint between the initial surface and the detected one, ensuring the reinjection occurs in the expected domain. Those corrected lengths can be formally written as $\delta_{Sc}$ and $\delta_{Fc}$:



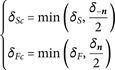



where $\delta_{n}$ and $\delta_{-n}$ are the distances between y and the next surface in direction ***n*** and −n respectively. These values of $\delta_{Sc}$ and $\delta_{Fc}$ are finally used instead of $\delta_{S}$ and $\delta_{F}$ to compute the probabilities *p*_*S*_, *p*_*F*_ and $p_{T_{i}}$ with Eq 20 (see Algorithm 5 in Appendix D).

## 5 Insensitivity to geometrical refinement

Before discussing the computation times, we need to describe how we make use of computer graphic tools for accelerating the line-surface intersections when geometrical description involves large numbers of geometrical primitives (triangles as far as we are concerned). We will be very brief because the very same approach is implemented, with the same libraries, in [1]. All required references and technical details can therefore be found in this reference. In short, before the simulation is launched, the geometry is processed (all the triangles defining the system boundary and solid-fluid interface) and a recursive grid is constructed that will be used each time a line-surface intersection is computed. This grid has no impact on the final result: it is only meant to improve the computation times. The main ideas are that the grid is locally refined so that the final elements include only a limited number of geometrical primitives (only a limited number of intersections need to be computed when entering these final elements), and the structure of the grid is designed to optimize data access in memory and to allow efficient out-of-core procedures when needed (when facing large geometrical data sets). The open-source libraries implementing these acceleration tools are star-engine [38] and embree [39].

A brief summary at this stage:

- thanks to double randomization, coupling is handled with simple successions of sub-paths dedicated to each physics (Sect 2),
- brownian walk algorithms are available that deal with confined domains with line-surface intersections only (Sect 3),
- and acceleration tools are available so that the computation of line-surface intersection will take approximately the same time for small or large numbers of geometrical primitives [1].

**Fig 5:**
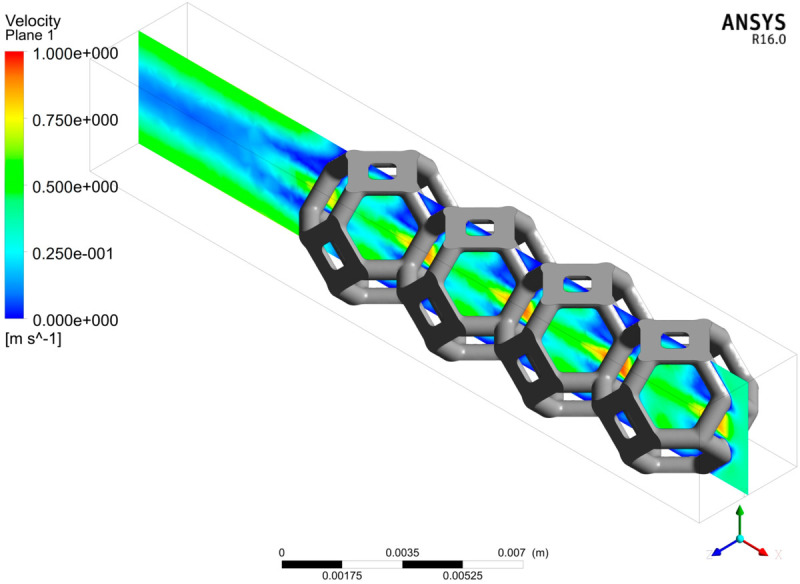
Stack of 4 kelvin cells and surface representation of the fluid flow for an homogeneous inlet fluid flow on the bottom right face and an outlet at the top left face. Fluid velocity at inlet face is 0.35m/s; maximum velocity is $1\;m.s^{-1}$. Pore size is 4mm and strand thickness is 0.7mm. This configuration is an emblematic example to study heat transfer (see Appendix F for the correspondences with such problems).

**Fig 6:**
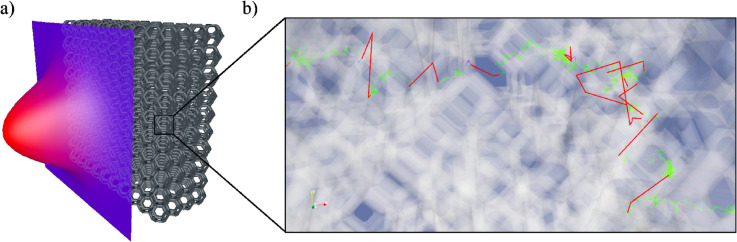
a) Representation of the deployed porous media submitted to an incident isotropic distribution function *f*_*i*_ with gaussian spatial profile at the inlet face. The gaussian profile ranges between $\frac{\eta_{min}}{4\pi }$ and $\frac{\eta_{max}}{4\pi }$. b) Representation of a typical path in the 3D geometry, which is constituted by three type of subpaths: advective subpaths (within the fluid) are in green, conductive subpaths (within the solid) in blue and linear transport subpaths (between interfaces, with possible absorption and scattering in the fluid) in red.

These are the three ideas that lead to the insensitivity that the present article aims to illustrate. We make this illustration using the porous structure of Figs 5 and 6. An elementary structure is set as a stack of four Kelvin cells in direction $e_{1}$ and is deployed in the two orthogonal directions $e_{2}$ and $e_{3}$ to form a solid foam that will be crossed by a flow. At the inlet face the flow is uniform, parallel to $e_{1}$. The three dimensional velocity field inside the foam is obtained numerically using a standard fluid mechanics solver. A symmetry condition is used for the lateral faces of the foam so that the velocity field is computed only once using the elementary structure (see Fig 5): the symmetry imposes that the flow remains the same in each stack of four Kelvin cells whatever the number of time this structure is deployed. We therefore define a porous geometry that can be enlarged by multiplying the number of pores as required, without the need to recompute the internal flow for each new configuration. As far as solid/fluid densities and the distribution function *f* are concerned, the same symmetry assumption is made at the four lateral faces of the deployed foam: null diffusive and advecto-diffusive fluxes, as well as specular reflection for linear transport. The inlet and outlet faces are shifted away from the foam (once the strand thickness at the inlet, twice the Kelvin-cell thickness at the outlet). At these two faces, the boundary conditions are therefore required only for the density in the fluid and the distribution function:

- the density $\eta_{F}$ in the fluid is uniform at a known value $\eta_{B}=\eta_{min}$ along the inlet face $\partial \Omega_{F}\;\cap \;{ℬ}_{D}$,
- the diffusion flux in the fluid is null at the outlet and lateral faces $\partial \Omega_{F}\cap {ℬ}_{R}$ (*e.g.* at the outlet face, the gradient $\nabla \eta_{F}.e_{1}$ for the density $\eta_{F}$ is null in the $e_{1}$ direction),
- the distribution function *f* for incoming directions at the inlet face is isotropic at $f_{i}=\frac{\eta_{min}}{4\pi }\;+\;\left(\frac{\eta_{max}}{4\pi }-\frac{\eta_{min}}{4\pi }\right)exp\left(-\frac{{\vec{y}}^{2}}{2\sigma^{2}}\right)$ where $\vec{y}$ is the 2D center position on inlet face and *σ* is five times the size of a single pore (see Fig 6),
- incoming distribution function at the outlet is uniform and isotropic at $f_{i}=\frac{\eta_{min}}{4\pi }$,
- incoming distribution function on the lateral faces is provided by specular reflection boundary conditions,
- the diffusion flux in the solid is null at the lateral faces $\partial \Omega_{S}\cap {ℬ}_{R}$ (there is no solid at the inlet and outlet faces, *i.e.* $\partial \Omega_{S}\cap {ℬ}_{D}=\emptyset$).

**Fig 7:**
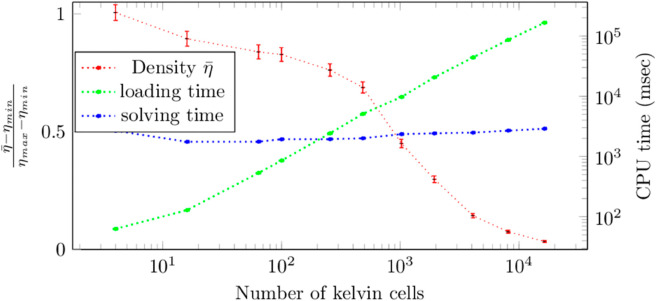
Evolution of the averaged outlet density $\bar{\eta }$ and computation time for an increasing number of kelvin cells. Loading time corresponds to the time required to generate and copy the geometry in the RAM memory as well as to build the acceleration grid, whilst the solving time is the time required to sample 10^4^ coupled random paths in the system. Diffusion coefficients are $D_{F}=D_{S}=5\;10^{-5}$, particles speed is c=0.05m/s, surface absorptivity is α=1, single-scattering and absorption frequencies are $\nu_{s}=\nu_{a}=12.5\;s^{-1}$, the single-scattering phase function is isotropic, *viz.* $p_{s}=\frac{1}{4\pi }$. Loading and computation times are obtained with an Intel(R) Core(TM) i9-9880H CPU @ 2.30GHz CPU.

Appendix F.4 provides an additional validation for the numerical schemes of Sect 3 using one single stack of four Kelvin cells and comparing the Monte Carlo estimates to a full simulation made with a standard deterministic solver. In Fig 7 we display Monte Carlo estimates of the average outlet density $\bar{\eta }$ (*i.e* the mean density in the fluid at the exit of the system), that start from this four-cells configuration and increase the deployment up to more than ten thousand cells. The problem is truly three-dimensional because of the gaussian-shape incident distribution function *f*_*i*_ at the inlet. Typical solving times for a one percent accuracy are 10*s* using a standard laptop, but as announced in introduction, the main result here is the fact that this computation time remains the same whatever the number of cells. Of course, as announced at the end of Sect 2, the computation time remains a decreasing function of the Knudsen number (or equivalently, an increasing function of the optical thickness).

## 6 Conclusion

In the present paper, we tackle the issue of the numerical handling of couplings between diffusion, advection and linear transport in complex geometries. The coupled problem is addressed in the same Monte Carlo terms as for a single physics: the solution of the problem is the expectancy of a random variable attached to a single random path. We combine the different physics (linearly) by dividing this main path into subpaths, each one describing a specific mode of transport. Doing so, we preserve the main idea of starting from the observation location (a camera in computer graphics, the outlet in the example above) and following a random path that visits the system until it reaches the sources. We still construct therefore, in a backward manner (with an adjoint approach), how the sources propagate throughout the system to contribute to the observation, but, with coupling, the path is more elaborate than for linear transport, with subpaths initiated one after the other.

We show that this approach preserves one on the most remarkable feature of Monte Carlo resolution of linear transport: insensitivity to geometrical refinement of the scene. To be achievable, this insensitivity requires each of the three items listed in the previous section: i) double randomization that enables the switch from one subpath to another, ii) advecto-diffusive walks algorithms compatible with confined domains requiring line-surface intersection only, and iii) acceleration tools developed in the field of computer graphics that make the computation of line-surface intersections insensitive to the number of geometrical primitives.

This insensitivity means that if we leave aside the modification of the physics itself (i.e. the paths have similar structures in each simulation) then the computation time is the same whatever the number of triangles. We insisted on the second item: this is because we managed to design path-sampling algorithms for brownian walkers in confined spaces using only the geometrical computation of line-triangle intersections. This could very well be revisited if the ongoing computer graphics research on sphere-triangle intersections reaches the same maturity as for line-triangle intersections. At present, we needed this step of slightly revisiting Brownian walks before we could benefit of the third essential point: the use of acceleration structures that are such that once the geometry has been loaded in memory, the computation times associated to the sampling of the path is strictly identical with four or several thousand cells. Altogether, we reach the very same conclusions as those of [1] concerning the handling of heavily detailed volume-descriptions, only now the physics is not restricted to radiative transfer. This enables to run simulation of engineering problems in complex geometries, that can be modified at will as the algorithms are independent of the geometry, which is just handled *via* line-surface intersection procedures. This feature paves the way to optimization procedures when it comes to defining the shape of the system itself.

In what we have presented here, there is however a strong restriction associated to the linearity of each of the considered phenomenologies and the linearity of their coupling: this is the condition under which double randomization allows the definition of single paths dealing with the coupled problem. Recent propositions [40] pave the way to address nonlinear physics with the same insensitivity property, but for now this results in branching paths statistics [41,42], whose management require a dedicated work.

We also insisted on the computational costs of multiple scattering in the low Knudsen number regime associated to such Monte Carlo algorithms. We could have also illustrated an increase of the computational cost when increasing the number of Kelvin cells along the foam thickness in Sect 5: dealing with 5 cells instead of 4 in this direction would have increased the computation times. There is something like a "coupling-Knudsen number" that plays a role similar to the Knudsen number and that is intrinsic to today’s Monte Carlo simulations. Handling this high-scattering regime remains out of scope of the present paper. However, whatever the geometry, we would still observe an insensitivity to the transversal deployment of the foam.

## Appendix

## A Simulation examples involving diffusion/linear-transport paths

An infinite uniform static fluid is considered (no drift) for which the fluid density $\eta_{F}$ is known outside a rectangular cuboid. The addressed quantity is $\eta_{F}$ at a given location $x_{obs}$ inside the cuboid. The algorithm is the one from Sect 2 (illustrated in Fig 2): a brownian walk starts at $x_{obs}$ and is interrupted exponentially in time; at this interruption location a linear-transport path starts until absorption; at the absorption location a new brownian walk is started, etc, until one of the brownian walks is interrupted outside the rectangular cuboid where $\eta_{F}$ is known; the Monte Carlo weight is the value of $\eta_{F}$ at this final location and $\eta_{F}(x_{obs})$ is estimated as the average of *N* such weights. Table 1 provides simulation examples for the following conditions:

- The faces of the cuboid are perpendicular to each of the unit vectors of an orthonormal basis $\left(e_{1},e_{2},e_{3}\right)$, the two extreme summits are (0,0,0) and (*L*,2*L*,3*L*), and the observation location is $x_{obs}=(L/2,L/2,L/2)$.
- For x outside the cuboid $\eta_{F}(x)=a\cdot x\;+\;b$ with $a=(\frac{2}{3L^{4}},\frac{2}{3L^{4}},\frac{2}{3L^{4}})$ and $b=\frac{1}{L^{3}}$.
- The absorption Knudsen number $\frac{c}{\nu_{a}L}$ is 1.
- The single scattering phase function is Heiney-Greenstein phase function with asymmetry parameter *g* = 0.5.
- The scattering Knudsen number $\frac{c}{\nu_{s}L}$ is varied from 0.01 to 100.
- The dimensionless diffusion coefficient $\frac{D_{F}}{cL}$ is varied from 0.01 to 100.
- The number of Monte Carlo samples is *N* = 10^5^.

In this configuration, the exact solution is $\eta_{F,exact}(x_{obs})=\frac{2}{L^{3}}$, independently of Knudsen number $\frac{c}{\nu_{s}L}$ and dimensionless diffusion coefficient $\frac{D_{F}}{cL}$. On the other hand, both the number of required samples for a 1% accuracy and the computation time depend on these parameters. The computation time is notably sensitive to the scattering Knudsen number (each multiple-scattering path involve more scattering events when decreasing $\frac{c}{\nu_{s}L}$) and to the dimensionless diffusion coefficient (when lowering $\frac{D_{F}}{cL}$ each diffusive/linear-transport path involves more diffusion/linear-transport switches before exiting the cuboid).

## B Trilinear profile

Let the trilinear profile $\eta (x^{\prime })=a\cdot x^{\prime }\;+\;b$ be the local expression of the density inside a sphere $𝒮(x,\delta_{S})$ of radius $\delta_{S}$ around x and let us check that Eq 14 gives exactly the density at x when using the adaptative step δ(x,u) defined in Sect 4.1 (thanks to symmetry):


$$\int_{4\pi }\frac{1}{4\pi }du\;\eta (x\;+\;\delta (x,u)u)=\int_{2\pi }\frac{1}{2\pi }du\;\left[\frac{1}{2}\;\eta (x\;+\;\delta (x,u)u)\;+\;\frac{1}{2}\;\eta (x-\delta (x,-u)u)\right]\;=\int_{2\pi }\frac{1}{2\pi }du\;\frac{1}{2}\left(a.(x\;+\;\delta (x,u)u)\;+\;b\;+\;a.(x-\delta (x,-u)u)\;+\;b\right)\;=\int_{2\pi }\frac{1}{2\pi }du\;\frac{1}{2}(2a.x\;+\;2b\;+\;a.u(\underset{=0}{\underbrace{\delta (x,u)-\delta (x,-u)}}))\;=\int_{2\pi }\frac{1}{2\pi }du\;\eta (x)\;=\eta (x)$$

where the unit sphere is noted 4π and 2π is any hemisphere of that sphere.

## C A Patankar scheme including sources due to linear transport

In Sect 4.2 we proposed an approximate random walk with drift inspired of Patankar work for advection diffusion in confined domains, with a probability to switch to linear transport at each Patankar step. This appendix provides the developments leading to this set of probability. The resulting path-sampling algorithm is presented in the appendix D (see algorithm 3).

We address the following advection-diffusion equation with a source due to the coupling with linear transport (see Eq 2 where $-\nabla .j_{T}=-\nu_{a}\left(\eta -\int f\;du\right)$ according to Eq 3; we note $\eta_{T}=\int f\;du$ as in Sect 4.2):

$$\nabla \cdot j\;+\;\nu_{a}(\eta -\eta_{T})=0$$ (23)

where the flux density vector is

$$j=-D_{F}\nabla \eta \;+\;v\eta$$ (24)

Using a finite volume approach, the balance on a cubic volume element of edge *δ* centered at x writes

$$\sum_{k=1}^{6}J_{k}\;+\;\delta^{3}\nu_{a}(\eta (x)-\eta_{T}(x))=0$$ (25)

where the densities *η* and $\eta_{T}$ in the source term of Eq 23 have been taken uniform and equal to the densities η(x) and $\eta_{T}(x)$ at the center of the volume element.

The fluxes *J*_*k* = 1,2,3,4,5,6_ through the 6 faces of the cube are approximated according to Patankar approach, that uses the analytic solution of one dimensional advection-diffusion equations −∇.j=0. 6 independent one-dimensional equations are constructed by projecting the flux density vector on the outward-pointing normals $u_{k=1,2,3,4,5,6}$ on each face of the cube:

$$-\nabla \cdot (j\cdot u_{k})\cdot u_{k}=0$$ (26)

with Dirichlet boundary conditions at x and $x\;+\;\delta u_{k}$ (*η* is known). The solution of this equation is

$$\eta (x\;+\;\sigma u_{k})=\eta (x)\;+\;\frac{\exp\left({\text{Pe}}_{k}\frac{\sigma }{\delta }\right)-1}{\exp({\text{Pe}}_{k})-1}(\eta (x\;+\;\delta u_{k})-\eta (x)),\;\sigma \in [0,\delta ]$$ (27)

with the Peclet numbers ${\text{Pe}}_{k}=\frac{v.u_{k}\;\delta }{D_{F}}$. The projections $j\cdot u_{k}$ of the flux density vector (see Eq 24) are obtained by differentiating Eq 27 with respect to *σ* to write the diffusion term $-D_{F}\nabla \eta \cdot u_{k}=-D_{F}\frac{\partial \eta }{\partial \sigma }$ and by substituting Eq 27 into the advection term $v\cdot u_{k}\eta$:

$$j\cdot u_{k}=v\cdot u_{k}\left(\eta (x)-\frac{\eta (x\;+\;\delta u_{k})-\eta (x)}{\exp({\text{Pe}}_{k})-1}\right)$$ (28)

This expression is used in the balance Eq 25, assuming uniform flux densities on each face of the cubic volume element equal to the flux density at the center of the face ($J_{k}=\delta^{2}j\cdot u_{k}$):

$$\sum_{k=1}^{6}\left[{\text{Pe}}_{k}\left(\eta (x)-\frac{\eta (x\;+\;\delta u_{k})-\eta (x)}{\exp\left({\text{Pe}}_{k}\right)-1}\right)\right]\;+\;\frac{\delta^{2}\nu_{a}}{D_{F}}(\eta (x)-\eta_{T}(x))=0$$ (29)

Finally, we choose to orient the cubic volume element such that $u_{1}=-v/|v|$ and $u_{2}=v/|v|$, leading to :

$${\text{Pe}}_{1}=-\text{Pe}$$ (30)

$${\text{Pe}}_{2}=\text{Pe}$$ (31)

$${\text{Pe}}_{3}={\text{Pe}}_{4}={\text{Pe}}_{5}={\text{Pe}}_{6}=0$$ (32)

where $\text{Pe}=\frac{||v||\;\delta }{D_{F}}$. Doing so (and using $\lim_{P_{e}\to 0}\frac{\text{Pe}}{\exp\left(\text{Pe}\right)-1}=1$) we obtain the following expression for η(x):

$$\eta (x)=p_{T}\;\eta_{T}(x)\;+\;(1-p_{T})\sum_{i=1}^{6}p_{i}\;\eta (x\;+\;\delta u_{i})$$ (33)

with

$$p_{T}=\frac{\frac{\delta^{2}\nu_{a}}{D_{F}}}{4\;+\;\frac{\text{Pe}}{\exp(\text{Pe})-1}\;+\;\frac{\text{Pe}}{1-\exp(-\text{Pe})}\;+\;\frac{\delta^{2}\nu_{a}}{D_{F}}}$$ (34)

$$p_{1}=\frac{\frac{\text{Pe}}{1-\exp\left(-\text{Pe}\right)}}{4\;+\;\frac{\text{Pe}}{\exp\left(\text{Pe}\right)-1}\;+\;\frac{\text{Pe}}{1-\exp\left(-\text{Pe}\right)}}$$ (35)

$$p_{2}=\frac{\frac{\text{Pe}}{\exp(\text{Pe})-1}}{4\;+\;\frac{\text{Pe}}{\exp\left(\text{Pe}\right)-1}\;+\;\frac{\text{Pe}}{1-\exp\left(-\text{Pe}\right)}}$$ (36)

$$p_{3}=p_{4}=p_{5}=p_{6}=\frac{1}{4\;+\;\frac{\text{Pe}}{\exp\left(\text{Pe}\right)-1}\;+\;\frac{\text{Pe}}{1-\exp\left(-\text{Pe}\right)}}$$ (37)

Working on the ratios of exponential functions, those probabilities can be reformulated as in Eq 16.

## D Algorithms

This appendix gathers all the propositions of Sect 4 under an algorithmic form. These algorithms are those used in Sect 5.

**Algorithm 1. Monte Carlo algorithm used to evaluate η(x).**



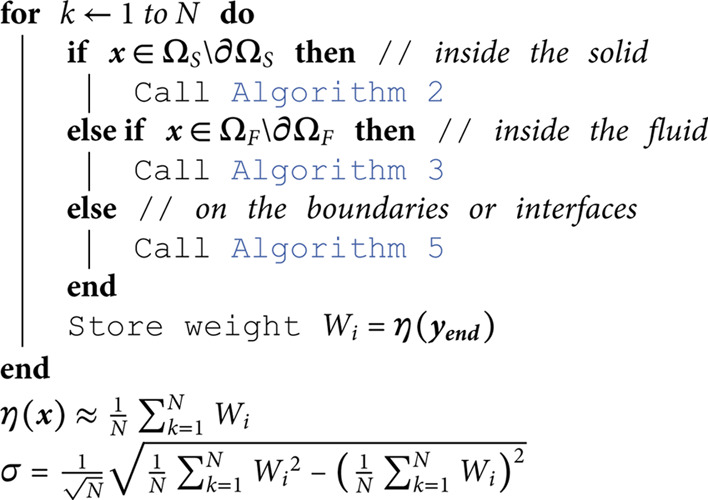



**Algorithm 2. Random walk for diffusion starting from point x in $\Omega_{S}\backslash \partial \Omega_{S}$ (see Sect 4.1)**



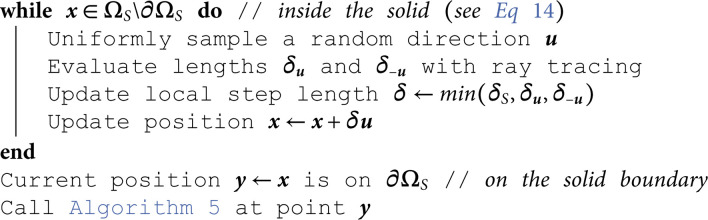



**Algorithm 3. Random walk for advection-diffusion coupled with linear transport, starting from point x in $\Omega_{F}\backslash \partial \Omega_{F}$ (see Sect 4.2).**



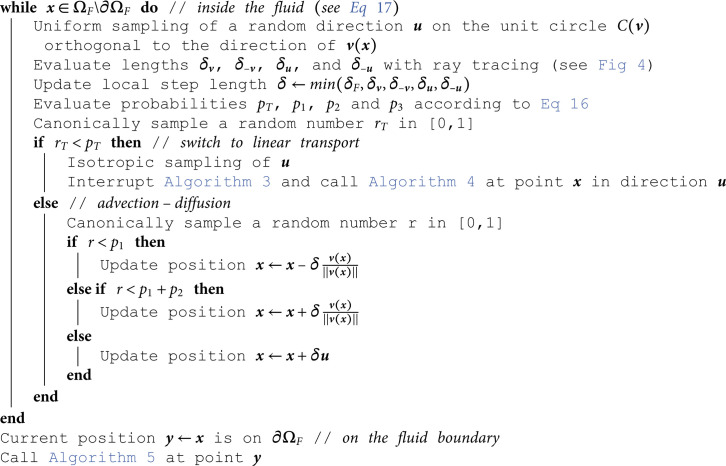



**Algorithm 4. Random walk for linear transport starting from point x in $\Omega_{F}\cup \partial \Omega_{F}$, in direction ***u***.**



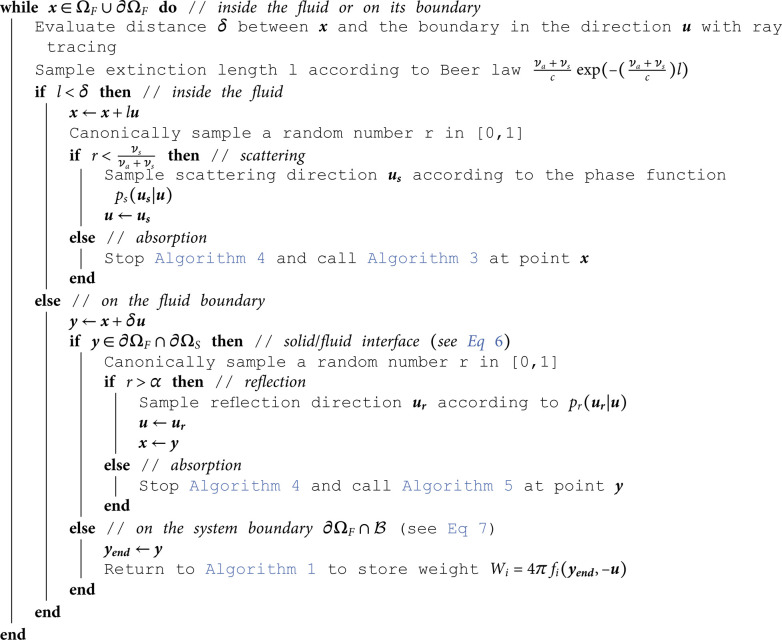



**Algorithm 5. Random walk for boundary conditions starting from point y on $\partial \Omega_{S}\cup \partial \Omega_{F}$ with normal ***n*** (see Sect 4.3).**



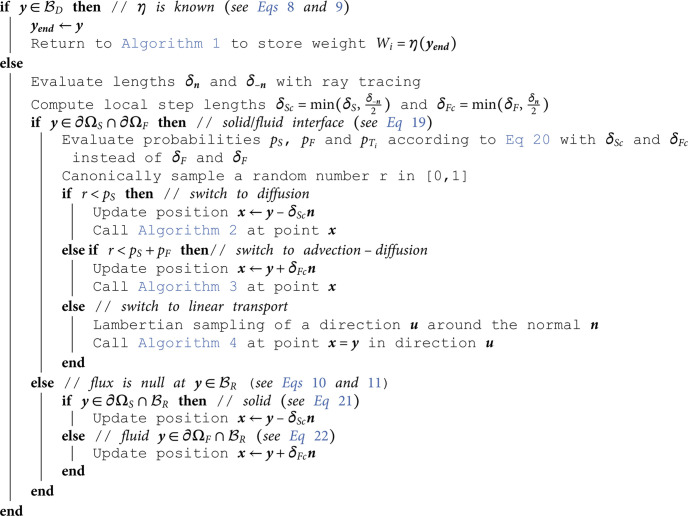



## E Validation

### E.1 Validation of the proposed random path compatible with ray tracing against an analytic solution for pure diffusion

Let us consider the following boundary value problem :

Δη(x)=0,x∈Ω (38)

$$\eta (y)=(y.e_{1}{)}^{2}\;+\;(y.e_{2}{)}^{2}-2(y.e_{3}{)}^{2}\;+\;y.e_{1}\;+\;y.e_{2}\;+\;y.e_{3}\;+\;1,\;y\in \partial \Omega$$ (39)

for which Eq 39 is solution everywhere in the field.

Results in Table 2 are obtained with Algorithm 1, along with Algorithms 2 and 5 (where we always have $y\in {ℬ}_{D}$ here), in the case where Ω is the unit cube defined by ∀x∈Ω, $0\leq x.e_{i}\leq 1$ where $\left(O,e_{1},e_{2},e_{3}\right)$ is an orthonormal basis.

**Table 2:** Results of Algorithms 1, 2, and 5 for N=10000 random samples on an analytic boundary value problem η(x) in a unit cube for various values of position $x.e_{1}=x.e_{2}=x.e_{3}=x=\frac{\left||x|\right|}{\sqrt{3}}$ and step length $\delta_{S}$. Monte Carlo results are shown in column $\tilde{\eta }$ along with their corresponding standard error *σ*.

$\delta_{S}$	*x*	η(x)	$\tilde{\eta }(x)$	σ
0.5	0.1	1.3	1.297	0.0026
0.5	0.3	1.9	1.889	0.0077
0.5	0.5	2.5	2.479	0.0109
0.5	0.7	3.1	3.099	0.0104
0.5	0.9	3.7	3.706	0.0053

0.1	0.1	1.3	1.302	0.0025
0.1	0.3	1.9	1.895	0.0075
0.1	0.5	2.5	2.499	0.0105
0.1	0.7	3.1	3.097	0.0101
0.1	0.9	3.7	3.705	0.0051
0.05	0.1	1.3	1.301	0.0025
0.05	0.3	1.9	1.907	0.0075
0.05	0.5	2.5	2.503	0.0106
0.05	0.7	3.1	3.097	0.0101
0.05	0.9	3.7	3.702	0.0051

These results indicate good convergence even for step lengths as big as half of the domain’s size (*i.e.* $\delta_{S}=0.5$). Such a good convergence is granted by the simplicity of the geometry of the domain Ω along with relatively smooth Dirichlet conditions on ∂Ω. In general, a step length of about 1/20th of the domain’s size should be retained in order to ensure a minimum convergence (*i.e.* $\delta_{S}=0.05$).

### E.2 Validation of the random path compatible with ray tracing against an analytic solution for advection-diffusion

Let us consider the following boundary value problem :

Δη(x)−v(x).∇η(x)=0,x∈Ω (40)

$$v(x)=||v(x)||\;e_{1}=-4\;e_{1},\;x\in \Omega$$ (41)

$$\eta (y)=(y.e_{2}{)}^{2}\;+\;(y.e_{3}{)}^{2}-y.e_{1}\;+\;y.e_{2}\;+\;y.e_{3}\;+\;1,\;y\in \partial \Omega$$ (42)

for which Eq 42 is solution everywhere in the field.

Results in Table 3 are obtained with Algorithm 1, along with Algorithms 3 and 5 (where we always have $y\in {ℬ}_{D}$ here), in the case where Ω is the unit cube defined by ∀x∈Ω, $0\leq x.e_{i}\leq 1$ where $\left(O,e_{1},e_{2},e_{3}\right)$ is an orthonormal basis.

**Table 3:** Results of Algorithms 1, 3, and 5 for N=10000 random samples on an analytic boundary value problem η(x) in a unit cube for various values of position $x.e_{1}=x.e_{2}=x.e_{3}=x=\frac{\left||x|\right|}{\sqrt{3}}$ and step length $\delta_{F}$. Monte Carlo results are shown in column $\tilde{\eta }$ along with their corresponding standard error *σ*.

$\delta_{F}$	*x*	η(x)	$\tilde{\eta }(x)$	σ
0.5	0.1	1.12	1.123	0.0029
0.5	0.3	1.48	1.474	0.0077
0.5	0.5	2	2.005	0.0094
0.5	0.7	2.68	2.682	0.0082
0.5	0.9	3.52	**3.533**	**0.0040**
0.1	0.1	1.12	1.117	0.0028
0.1	0.3	1.48	1.476	0.0078
0.1	0.5	2	2.000	0.0098
0.1	0.7	2.68	2.675	0.0085
0.1	0.9	3.52	3.525	0.0040
0.05	0.1	1.12	1.116	0.0027
0.05	0.3	1.48	1.490	0.0078
0.05	0.5	2	2.005	0.0098
0.05	0.7	2.68	2.671	0.0085
0.05	0.9	3.52	3.522	0.0041

By comparison with the pure diffusion example of Sect E.1, present results indicate good convergence but not for step lengths as big as half of the domain’s size anymore (see result in red). $\delta_{F}$ plays therefore the role of standard numerical parameter for which convergence must be specifically tested (the value of 1/20th of the domain’s size suggested above for pure diffusion can not be retained as such).

### E.3 Trade-offs between accuracy and computational efficiency

#### E.3.1 Discussion on the Bias introduced by the step size and wall re-injection in random walks.

To begin this discussion, it is helpful to distinguish between two types of random walks used in this work: those modeling pure linear transport and those representing advective-diffusive phenomena. As already stated, random walks for linear transport are strictly unbiased, and the associated Monte Carlo methods are well-established and robust.

More fundamentally, it is important to clarify that there are two distinct sources of bias, which are not always clearly separated in the literature:

1. A discretization bias, which arises when the original physical equations are numerically approximated using finite-difference schemes or equivalent operators. This bias is independent of the Monte Carlo method itself and reflects a modeling approximation.
2. A statistical bias, which may occur if the Monte Carlo method fails to produce an exact estimator of the target (discretized or continuous) equations. This occurs when the sampling method does not yield a statistically consistent estimator of the equation being solved.

When we refer to an unbiased random walk, we mean that neither of these two biases is present: the method both exactly represents the physical equations and provides a statistically exact Monte Carlo estimator.

Returning to the advective-diffusive case: bias-related issues must be treated differently depending on whether the walk occurs in the interior of the domain or originates from the boundary. In certain idealized conditions (such as uniform velocity and thermophysical fields) it is possible to construct unbiased interior walks (e.g., using WOS combined with Von Mises-Fisher directional sampling). However, generating unbiased walks from boundaries is substantially more challenging.

Given the generality of the method we propose in this article, we choose to accept the presence of discretization-related bias in both the interior walks and the wall re-injection mechanism. In practical terms, the bias introduced by the step size (whether in the bulk or at the boundary) can be interpreted through the lens of finite-difference approximations. We refer the reader to [11] which shows a rigorous equivalence between the bias of random walks with fixed step size *δ* and the discretization error $\delta_{FD}$ from finite-difference schemes with $\delta_{FD}≃\delta$.

In this sense, the bias we refer to is essentially that of a finite-difference representation of the advective-diffusive operator. Importantly, the Monte Carlo estimator we construct remains statistically unbiased with respect to this discretized formulation. This interpretation places our method within the established framework of deterministic numerical schemes, and thus allows for the use of standard numerical analysis techniques, including truncation error estimation and convergence studies, as commonly applied to finite-difference schemes.

#### E.3.2 Computational performance as a function of step size.

The question of computational performance cannot, of course, be separated from the issue of bias discussed above. Indeed, achieving an acceptable level of bias, compatible with the desired accuracy constraints, requires choosing a random walk step that is sufficiently small relative to the physical and geometric features of the scene. In practice, this means that the step must be small enough to resolve the relevant characteristic lengths, whether they originate from geometry (such as curvature, obstacles, or interfaces) or from physical considerations (such as gradients in state variables or spatial variations in boundary conditions and source terms within the volume). Ensuring that the random walk step is adequate to capture these spatial variations with sufficient precision is essential for both accuracy and reliability of the simulation.

This naturally leads to the question of computation time, given the choice of a specific random walk step. To properly address this issue, it is essential to distinguish between two separate aspects. The first concerns the computational cost associated with each individual step, particularly the geometric intersection tests with the scene. The second relates to the estimation of the average number of steps required per path within the domain.

The first aspect is discussed elsewhere in the text, where we show that techniques originally developed in the context of image synthesis and computer graphics provide highly efficient tools for performing intersection queries in a manner that is largely insensitive to geometric complexity.

The second aspect, which concerns estimating the average number of steps per walk, is a significantly more subtle problem rooted in physics and mathematics. It is generally studied under the notion of first-passage times to the boundary, which may refer either to the first exit from an interior point or to the first return to the boundary after a re-injection.

There exist, in this context, both well-established classical results and more subtle findings that have emerged from recent work, particularly in the field of statistical physics. For example, in the case of purely diffusive transport or for Boltzmann walkers as described earlier in this paper, the scaling behavior of the mean number of steps is well known. The reader interested in deeper or more complex behaviors may refer to discussions such as those in [44–46]. In typical diffusive regimes, whether the underlying walk is Brownian-like or of Boltzmann type with a small mean free path, it is straightforward to show that the mean number of steps required to reach the boundary scales as $1/\delta^{2}$, where *δ* is the random walk step. Consequently, reducing the step size by one order of magnitude results in an approximate hundredfold increase in the total number of steps, and thus in computation time.

However, it is crucial to recognize that this specific scaling, while serving as a useful guide in many standard situations, does not directly apply in full generality to the problems we address here. A more problem-specific analysis is necessary, due in particular to the following features:

- When the walk reaches a boundary, it may continue on either side, depending on a re-injection rule governed by the physical properties of the adjacent media.
- Each complete path consists of segments alternating between advective-diffusive motion and pure linear transport, with the transition probabilities between regimes also determined by the physical properties of the materials involved.
- The interplay between advection and diffusion can produce highly nontrivial first-passage characteristics. For instance, in the case of a strongly oriented, essentially one-dimensional flow with dominant advection, the number of steps required to reach a boundary may remain relatively small. In contrast, in flow configurations dominated by recirculation, such as vortices or closed streamlines, advection can trap the walker and the escape becomes possible only through diffusion.

In summary, it is essential to perform a dedicated analysis for each specific problem to estimate the mean number of steps per path. Fortunately, the physicist familiar with the structure of the system under consideration often possesses enough insight to derive reasonable estimates of these quantities, and thus to anticipate how a change in the random walk step may affect computation time.

## F Application to heat transfer

The model studied in the body of the article is quite general and can be applied to different applicative fields, *e.g.* neutron transport, charge carrier transport in semiconductors *etc.*, as discussed in introduction. Among these potential applications, here we detail how the present work can be directly used to address heat transfer problems.

The example in Sect 5 is directly inspired of an academic porous exchanger from the heat transfer literature [43]. The main assumption in such standard heat transfer applications is that radiative transfer can be linearized as function of temperature, which imposes that the relative temperature differences remain limited. This assumption is detailled in F.1. F.2 provides the resulting correspondences between the model addressed in the body of the article and heat transfer. F.3 and F.4 provide numerical solutions, obtained from standard heat transfer solvers, that are used for validation.

### F.1 Radiance temperatures

We start from the stationary radiative transfer equation formulated in terms of monochromatic specific intensity $I_{\nu }\equiv I_{\nu }(\vec{x},\vec{u},t)$ at position $\vec{x}$, in direction $\vec{u}$ at time *t* and frequency ν :

$$\vec{u}.\vec{\nabla }I_{\nu }=-k_{a}^{\nu }I_{\nu }\;+\;k_{a}^{\nu }I_{\nu }^{eq}-k_{s}^{\nu }I_{\nu }\;+\;k_{s}^{\nu }\int_{4\pi }p_{s,\nu }(\vec{u}|{\vec{u}}^{\prime })I_{\nu }^{\prime }du^{\prime }$$ (43)

where $k_{a}^{\nu }$ is the absorption coefficient, $k_{s}^{\nu }$ the scattering coefficient, $p_{s,\nu }$ the scattering phase function, $I_{\nu }^{\prime }\equiv I_{\nu }(\vec{x},{\vec{u}}^{\prime },t)$ and $I_{\nu }^{eq}\equiv I_{\nu }^{eq}(\theta (\vec{x},t))$ the specific equilibrium intensity at temperature $\theta (\vec{x},t)$ within the medium. Although the radiative transfer is stationary, $I_{\nu }$ depends on *t* due to the evolution of the solid and fluid temperature.

Assuming that for all times and positions, the solid and fluid temperature remains close to a reference temperature $\theta_{ref}$, the temperature dependence of the specific equilibrium intensity can be linearized:

$$I_{\nu }^{eq}(\theta )\approx I_{\nu }^{eq}(\theta_{ref})\;+\;\partial_{\theta }I_{\nu }^{eq}(\theta_{ref})(\theta -\theta_{ref})$$ (44)

Moreover, equilibrium properties allow to write :

$$0=-k_{a}^{\nu }I_{\nu }^{eq}\;+\;k_{a}^{\nu }I_{\nu }^{eq}-k_{s}^{\nu }I_{\nu }^{eq}\;+\;k_{s}^{\nu }\int_{4\pi }p_{s,\nu }(\vec{u}|{\vec{u}}^{\prime })I_{\nu }^{eq}du^{\prime }$$ (45)

Introducing the notation ${\tilde{I}}_{\nu }=I_{\nu }\;-\;I_{\nu }^{eq}(\theta_{ref})$ for the perturbations and subtracting Eqs (43) and (45), the radiative transfer equation under the assumption (44) can be written as follows:

$$\vec{u}.\vec{\nabla }{\tilde{I}}_{\nu }\approx -k_{a}^{\nu }{\tilde{I}}_{\nu }\;+\;k_{a}^{\nu }\partial_{\theta }I_{\nu }^{eq}(\theta_{ref})(\theta -\theta_{ref})-k_{s}^{\nu }{\tilde{I}}_{\nu }\;+\;k_{s}^{\nu }\int_{4\pi }p_{s,\nu }(\vec{u}|{\vec{u}}^{\prime }){\tilde{I}}_{\nu }^{\prime }du^{\prime }$$ (46)

We choose to rewrite this equation using the radiance temperature $\theta_{R,\vec{u}}^{\nu }$ in the direction $\vec{u}$. This radiance temperature is a spectral and directional quantity defined as the temperature for which the equilibrium specific intensity is equal to the specific intensity:

$$I_{\nu }^{eq}(\theta_{R,\vec{u}}^{\nu }(\vec{x},t))=I_{\nu }(\vec{x},\vec{u},t)$$ (47)

Using Eq 44 to express $I_{\nu }^{eq}(\theta_{R,\vec{u}}^{\nu }(\vec{x},t))$,

$$I_{\nu }\approx I_{\nu }^{eq}(\theta_{ref})\;+\;\partial_{\theta }I_{\nu }^{eq}(\theta_{ref})(\theta_{R,\vec{u}}^{\nu }-\theta_{ref})$$ (48)

and therefore,

$${\tilde{I}}_{\nu }\approx \partial_{\theta }I_{\nu }^{eq}(\theta_{ref})(\theta_{R,\vec{u}}^{\nu }-\theta_{ref})$$ (49)

Eq 46 becomes :

$$\vec{u}.\vec{\nabla }\theta_{R,\vec{u}}^{\nu }\approx -k_{a}^{\nu }\theta_{R,\vec{u}}^{\nu }\;+\;k_{a}^{\nu }\theta -k_{s}^{\nu }\theta_{R,\vec{u}}^{\nu }\;+\;k_{s}^{\nu }\int_{4\pi }p_{s,\nu }(\vec{u}|{\vec{u}}^{\prime })\theta_{R,{\vec{u}}^{\prime }}^{\nu }du^{\prime }$$ (50)

For didactic reasons we here make the assumption of grey absorbing and scattering fluid (there would be no difficulty associated to the full preserving of spectral dependancies), which writes :

$$\vec{u}.\vec{\nabla }\theta_{R,\vec{u}}\approx -k_{a}\theta_{R,\vec{u}}\;+\;k_{a}\theta -k_{s}\theta_{R,\vec{u}}\;+\;k_{s}\int_{4\pi }p_{s}(\vec{u}|{\vec{u}}^{\prime })\theta_{R,{\vec{u}}^{\prime }}du^{\prime }$$ (51)

with $\theta_{R,\vec{u}}\equiv \theta_{R,\vec{u}}^{\nu }$ at all frequencies ν.

Similarly at the boundary :

$$\theta_{R,\vec{u}}=\alpha \theta \;+\;(1-\alpha )\int_{u^{\prime }.n(y)<0}p_{r}(u|u^{\prime })\;\theta_{R,{\vec{u}}^{\prime }}\;d{\vec{u}}^{\prime }$$ (52)

where *α* is the surface absorptivity (or emissivity), *θ* the surface temperature and $p_{r}(u|u^{\prime })$ the distribution for reflection directions.

Eqs 51 and 52 define a closed system for $\theta_{R,\vec{u}}$ from wich the volumic radiative power $\psi_{R}$ and the surfacic radiative power $\varphi_{R}$ write

$$\psi_{R}=-\zeta \left(\theta -\int_{4\pi }\frac{1}{4\pi }\theta_{R,\vec{u}}du\right)$$ (53)

and

$$\varphi_{R}=-h_{R}\left(\theta -\int_{u^{\prime }.n(y)<0}\frac{\left|\vec{u}.\vec{n}\right|}{\pi }\theta_{R,\vec{u}}du\right)$$ (53)

with $\zeta =16k_{a}\sigma \theta_{ref}^{3}$, $h_{R}=4\alpha \sigma \theta_{ref}^{3}$, *α* is the surface emissivity and *σ* the Stefan-Boltzmann constant.

### F.2 Correspondences of physical quantities

Table 4 provides the correspondences between the model addressed in the body of the article and heat transfer problems. Substituting these correspondences in the model Eqs 1–10 the resulting heat transfer model is:



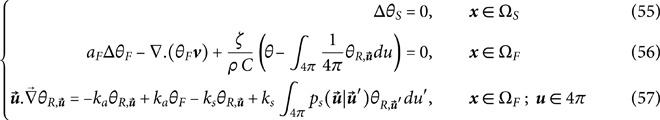



where we used $-\nabla .j_{T}=\psi_{R}$ (see Eq 53); $\theta_{S}$ and $\theta_{F}$ are the temperatures within the solid and the fluid respectively. At the solid-fluid interface,



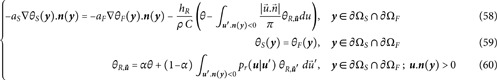



where we used $j_{T}(y).n(y)=\varphi_{R}$ (see Eq 53). At the boundary of the system,



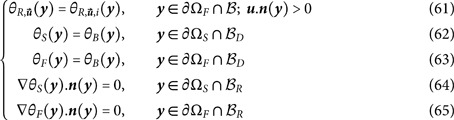



**Table 4:** Correspondences between the model addressed in the body of the article and heat transfer problems.

Model in Sect 1	Heat transfer problems
$\frac{\eta }{\rho C}$	*θ* (temperature); We choose $\rho_{F}C_{F}=\rho_{S}C_{S}=\rho C$ for the sake of simplicity (this is not a restriction of the approach)
$\frac{4\pi f}{\rho C}$	$\theta_{R,\vec{u}}$ (radiance temperature)
$\frac{\nu_{a}}{c}$	*k*_*a*_ (absorption coefficient)
$\frac{\nu_{s}}{c}$	*k*_*s*_ (scattering coefficient)
$\left(\frac{\rho Cc}{16\sigma }\right){\;}^{1/3}$	$\theta_{ref}$ (reference temperature for radiation linearisation); this correspondence is of application for the definition of the particles speed *c*.
*D*	$a=\frac{\lambda }{\rho C}$ (thermal diffusivity), where *λ* is the thermal conductivity.
Eq 1	Steady state heat equation for opaque solids.
Eq 2	Steady state advection-diffusion heat equation for semi-transparent fluids.
Eq 3	Radiative transfer Eq 51

Note that using the definition of the particles speed *c* as a function of $\theta_{ref}$ provided in Table 4, the probability set for path-switching at the boundaries in Eq 20 becomes



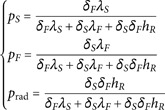



where $h_{R}=4\alpha \sigma \theta_{ref}^{3}$; $\lambda_{S}$ and $\lambda_{F}$ are the thermal conductivity for the solid and fluid respectively.

### F.3 Poiseuille duct

**Fig 8:**
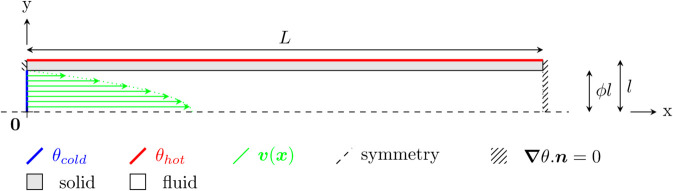
2D-slice of an isolated duct structure used as a heat exchanger between a thermal source at $\theta_{hot}$ and an incoming cold Poiseuille circulation at $\theta_{cold}$. Retained values for the simulation are: $\frac{L}{l}=10$, ϕ=0.8, where *ϕ* is the ratio of inner duct radius over outer radius, $\frac{\lambda_{S}}{\lambda_{F}}=10$, where $\lambda_{S}$ and $\lambda_{F}$ are the thermal conductivity for the solid and fluid respectively, and $\max\left(\frac{||v||l}{a_{F}}\right)=5$, where *a*_*F*_ is the thermal diffusivity for the fluid.

**Fig 9:**
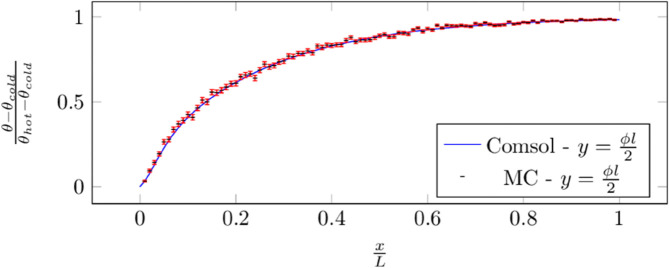
Poiseillle duct results. Comparison of the temperature obtained with Comsol Multiphysics and the Monte Algorithm for the configuration defined in Fig 8. In these simulations, $\delta_{S}=\delta_{F}=\frac{l}{100}$.

Algorithm 5 along with Algorithms 2 and 3 are used to solve the 2D configuration presented in Fig 8: a cold Poiseuille flow enters a 2D solid duct which is heated by its external faces. In this configuration, no radiation is taken into account. Results are compared with a *Comsol Multiphysics* simulation in Fig 9 for various positions in the domain. The number of Monte Carlo samples are kept at 1000 so that error-bars remain visible.

### F.4 Kelvin-cell porous heat exchanger

**Fig 10:**
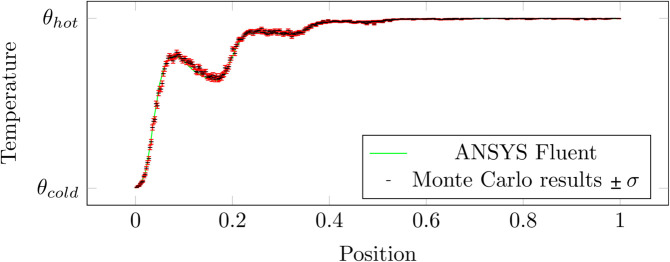
Comparison of the temperature obtained with ANSYS Fluent and the Monte Algorithm for the stack of 4 Kelvin cells presented in Fig 5. Results for locations on the center line going down the flow; the inlet face is at adimensionned position 0 and the outlet at adimensionned position 1. In order to obtain a curve showing high variations of temperature in the fluid, the solid’s and fluid’s thermal diffusivities are respectively set at $80.10^{-6}\;m^{2}.s^{-1}$ and $130.10^{-6}\;m^{2}.s^{-1}$. The inlet velocity field is uniform at $1\;m.s^{-1}$.

Algorithm 5 along with Algorithms 2, 3 and 4 are used to solve the 3D configuration presented in Fig 5: a solid stack of 4 kelvin cells is heated by a radiative temperature $\theta_{hot}$ set on the inlet face and cooled by the fluid at $\theta_{cold}$ which enters by the same face. On the outlet face, the radiative temperature is set at $\theta_{cold}$ to represent the environment. As in Sect 5, symmetry boundary conditions are used on the lateral faces. Radiative transfer is here reduced to only direct exchange between surfaces (the fluid is perfectly transparent, *i.e.* $\nu_{a}=\nu_{s}=0$). Results are compared with an *ANSYS Fluent* simulation in Fig 10.

## Supporting information

S1 File This contains the data used to draw Fig 7. (TXT)

S2 File This contains the data computed with Comsol Multiphysics that are represented in Fig 9. (TXT)

S3 File This contains the data computed with our Monte Carlo approach that are represented in Fig 9. (TXT)

S4 File This contains the data computed with ANSYS Fluent that are represented in Fig 10. (TXT)

S5 File This contains the data computed with our Monte Carlo approach that are represented in Fig 10. (TXT)
